# Development of a checklist to assess the quality of reporting of knowledge translation interventions using the Workgroup for Intervention Development and Evaluation Research (WIDER) recommendations

**DOI:** 10.1186/1748-5908-8-52

**Published:** 2013-05-16

**Authors:** Lauren Albrecht, Mandy Archibald, Danielle Arseneau, Shannon D Scott

**Affiliations:** 1 University of Alberta, Level 3, Edmonton Clinic Health Academy, Edmonton, AB, Canada

**Keywords:** Behavior change intervention reporting, Knowledge translation interventions, Reporting checklist, Quality assessment, Systematic review

## Abstract

**Background:**

Influenced by an important paper by Michie *et al.*, outlining the rationale and requirements for detailed reporting of behavior change interventions now required by Implementation Science, we created and refined a checklist to operationalize the Workgroup for Intervention Development and Evaluation Research (WIDER) recommendations in systematic reviews. The WIDER recommendations provide a framework to identify and provide detailed reporting of the essential components of behavior change interventions in order to facilitate replication, further development, and scale-up of the interventions.

**Findings:**

The checklist was developed, applied, and improved over the course of four systematic reviews of knowledge translation (KT) strategies in a variety of healthcare settings conducted by Scott and associates. The checklist was created as one method of operationalizing the work of the WIDER in order to facilitate comparison across heterogeneous studies included in these systematic reviews. Numerous challenges were encountered in the process of creating and applying the checklist across four stages of development. The resulting improvements have produced a ‘user-friendly’ and replicable checklist to assess the quality of reporting of KT interventions in systematic reviews using the WIDER recommendations.

**Conclusions:**

With journals, such as Implementation Science, using the WIDER recommendations as publication requirements for evaluation reports of behavior change intervention studies, it is crucial to find methods of examining, measuring, and reporting the quality of reporting. This checklist is one approach to operationalize the WIDER recommendations in systematic review methodology.

## Findings

### Current state of behavior change intervention studies

Behavior change interventions (BCIs) can be effective ways to improve health outcomes and cut health spending [1]. While expertise in the field of designing, evaluating, and implementing BCIs exists, a few key barriers hamper successful large-scale application of BCIs. Recent research suggests that interventions are only described in detail 5% to 30% of the time [2-5]. The result is that readers know few details about the components of interventions and the relationship between these components, which are responsible for observed changes or outcomes. It is well established that understanding the details of interventions and the relationships between intervention components is key to replicating BCIs, as well as further development and scale-up [6]. This article describes one solution to identify gaps in the reporting of intervention evaluation studies.

### Calls for standardized reporting of behavior change interventions

In 2001 the Consolidated Standards of Reporting Trials (CONSORT) group developed evidence-based recommendations to standardize reporting of randomized controlled trials (RCTs) [7]. With an emphasis on transparency, a 25-item checklist and flow diagram offered researchers a scientific method to prepare reports of RCT findings. In 2003, Davidson *et al.*[8] augmented the CONSORT Statement to address unique features of conducting behavioral medicine interventions in RCT studies. Further developments took place in 2008, with a publication authored by Abraham and Michie [9] that called for standardized language to describe BCIs, published standardized intervention protocols or manuals, and identification and description of links between behavior change techniques and theoretical frameworks of potential change mechanisms. Without these scientific advancements the ability of BCI evaluations to contribute to evidence-based practice is limited.

### Emergence of the WIDER recommendations

In 2007, following the 21^st^ annual conference of the European Health Psychology Society, the WIDER issued a consensus statement (Additional file 1). This statement synthesized earlier calls for standardized reporting of BCIs [8,9] and outlined a four-pronged method for BCI reporting to be used in tandem with the CONSORT statement [10]. The philosophy behind this publication was that greater clarity regarding the functional components of behavior change interventions is essential to ensure that interventions are delivered to influence outcomes. The WIDER recommendations (Table 1) are now an established framework to identify and describe the essential components for detailed reporting of BCIs. BCI publications are now required to apply the WIDER recommendation in their work in order to publish BCI studies in journals such as Implementation Science [6] and Addiction [11].

**Table 1:** WIDER recommendations to improve reporting of the content of behavior change interventions

**WIDER recommendations**	**Supplementary recommendations**
Detailed description of interventions in published papers	1) Characteristics of those delivering the intervention
	2) Characteristics of the recipients
	3) The setting
	4) The mode of delivery
	5) The intensity
	6) The duration
	7) Adherence/fidelity to delivery protocols
	8) Detailed description of the intervention content provided for each study group
Clarification of assumed change process and design principles	1) The intervention development
	2) The change techniques used in the intervention
	3) The causal processes targeted by these change techniques
Access to intervention manuals/protocols,	Submit protocols or manuals for publication to make these supplementary materials easily accessible (*i.e.*, online).
Detailed description of active control conditions	1) Characteristics of those delivering the control
	2) Characteristics of the recipients
	3) The setting
	4) The mode of delivery
	5) The intensity
	6) The duration
	7) Adherence/fidelity to delivery protocols
	8) Detailed description of the control content provided

### Employing the WIDER recommendations in our work

Given the current state of the science related to evaluating BCIs and the increasing importance of synthesizing literature in this field from a variety of research designs (*i.e.*, RCTs, observational studies, qualitative research, etc.), the purpose of this paper is to present one approach for systematically operationalizing the WIDER recommendations in systematic review methodology. This article describes the iterative process of creating, using, and improving a WIDER Recommendation Checklist and offers recommendations for its future development and use in systematic reviews.

### Conducting four systematic reviews: the impetus for the WIDER recommendations checklist

Since 2010, we (Scott and associates) have conducted four systematic reviews investigating knowledge translation (KT) interventions in a variety of healthcare professions and settings. KT interventions can be likened to BCIs; however, they are broader in scope and include professional, financial, organizational, and structural interventions [12]. The first systematic review investigated the use of KT interventions in the allied health professions (*i.e.*, dietetics, occupational therapy, pharmacy, physiotherapy, speech-language pathology) and was recently published in Implementation Science [13]. A second systematic review, currently in progress, focuses on KT interventions employed within the rehabilitation medicine professions (*i.e.*, a subset from the first systematic review). The third systematic review, also currently in progress, investigates the use of KT interventions to promote research uptake with pediatric healthcare professionals in child health settings. The fourth systematic review, currently in the data extraction phase, investigates the use of narrative and arts-based KT strategies in healthcare. These three systematic reviews have not yet been published.

### Rationale and development of the WIDER recommendations checklist

In order to be responsive to the needs of decision makers, the complexity of the clinical practice landscape, and the diversity of the KT literature, our systematic reviews are guided by an assumption of methodological inclusivity. Thus, a variety of study designs were included in order to capture, report, and synthesize diverse forms of evidence. Due to the broad scope of our systematic reviews, it was a challenge to compare KT interventions across a wide range of study designs, settings, and professions. The variety of interventions employed to effect diverse behavior changes and the various ways in which these changes were measured added further complexity to data synthesis. In order to facilitate comparison across heterogeneous studies included in each of the systematic reviews, we used the WIDER recommendations to examine the quality of the reporting of KT interventions in order to better understand and compare the types of interventions being implemented.

### Phase one of checklist development: using the WIDER recommendations to compare heterogeneous KT intervention evaluation studies

In our first attempt at applying the WIDER recommendations within the context of a systematic review, we used a general checklist in which the four WIDER recommendations were broadly applied to each KT intervention study (Additional file 2). In this early conceptualization of the checklist, all supplementary recommendations must have been described to satisfy each WIDER recommendation. While this was a novel approach for comparing diverse interventions based on the quality of KT intervention reporting, this method largely resulted in the four WIDER recommendations being classified as ‘not met’ across the studies. Additionally, by collapsing these supplementary recommendations into the four broad WIDER recommendations, we were unable to identify specific areas of deficient reporting within each the WIDER recommendations.

### Phase two of checklist development: refining the checklist by illustrating a gradient

In the second iteration of the checklist, we sought to measure the degree to which studies satisfied each of the WIDER recommendations in order to account for the aforementioned conceptual difficulties (Additional file 3). Each WIDER recommendation was segmented into the supplementary recommendations, which allowed for partial fulfillment of each recommendation. By reporting a gradient within each WIDER recommendation, we identified areas that were well reported as well as areas that were poorly reported across studies. This, allowed us to make recommendations to improve future reporting of KT intervention evaluation studies.

Working with the second version of the checklist was an iterative process involving frequent revisiting of previously extracted articles and working dialogue with team members. Developing a clear visual depiction of the second iteration of our WIDER recommendations Checklist, (*i.e.* including supplementary recommendations), was challenging as we sought to balance visual accessibility with comprehensiveness of reporting. We discussed various options for depicting the supplementary recommendations, such as a table format and a ‘thermometer’ representation. Ultimately, a Microsoft Excel spreadsheet with colored fonts and color ‘blocks’ was used to facilitate visual appeal of the checklist, while at the same time clearly depicting the degree to which each of the WIDER recommendations were met in each study.

The second version of the WIDER Recommendation Checklist required diligent and deliberate recognition of many components related to the intervention (*e.g.*, time of delivery, developmental basis for intervention) that could easily have gone unnoticed in the previous iteration of the checklist. It also provided insight into authors’ assumptions regarding interventions. For example, a lack of reporting in specific supplementary recommendations suggests that authors do not recognize these as important, or did not anticipate that deficient reporting in this area could influence the interpretation of study results. For example, demographic characteristics of intervention and control group participants were frequently not separated. Grouping this information together made it impossible to infer if differences between the intervention and control groups influenced outcomes. Additionally, deficient reporting for specific supplementary recommendations may point to confusion regarding the recipient of the intervention and the level or focus of the behavior change. For example, interventions delivered to the healthcare professional, but measured in terms of patient outcomes were difficult to evaluate.

The main challenge in applying the WIDER Recommendations Checklist stemmed from the lack of clear definitions regarding each of the four recommendations and their supplementary recommendations. These problems persisted despite frequent team discussions and it was difficult to ‘draw the line’ between a category being satisfied or not satisfied. Additionally, while partial fulfillment of a category was an improvement, it was difficult to visually portray the specific supplementary recommendations that were consistently described or not described across the studies.

### Phase three of checklist development: capturing the nuances of the WIDER recommendations

The third version of the WIDER Recommendations Checklist was developed to more accurately capture the specific supplementary recommendations within each of the WIDER recommendations that were consistently satisfied (or not satisfied) through the addition of a legend to the Microsoft Excel table (Additional file 4). This helped us to represent the nuances of each of the four WIDER recommendations and to clearly depict patterns of reporting. Developing this version of the checklist was a dynamic and iterative process, involving frequent team discussions and revisiting previously extracted articles. Our primary goal was to build on the strengths of our previous versions of the checklist and to address some of the limitations by creating a user-friendly version with visual impact. We believe that this strengthened our most recent systematic review by allowing us to compare each WIDER recommendation and the supplementary recommendation consistently and on an individual basis across the studies. Additionally, the format of this version of the checklist makes it easily replicable in other systematic reviews.

### Phase four of checklist development: developing a glossary and conducting reliability testing

We are currently developing a fourth version of the WIDER Recommendations Checklist to be used in our fourth systematic review. In this version, we are developing a glossary containing clear definitions and examples to guide decision making when using the checklist. We see this as essential to defining the scope of each WIDER recommendation and providing clear guidelines to determine whether a study has met each recommendation and the supplementary recommendations. The glossary will be used alongside the checklist and we will conduct reliability testing of the checklist to ensure that this quality assessment tool can be applied by other researchers conducting systematic reviews or KT intervention evaluation studies.

### Future development of the WIDER Recommendations checklist

While we support the usability of our WIDER Recommendations Checklist, we also acknowledge that improvements can be made. First, the checklist could be studied for validity. Second, we believe that determining a method of ‘weighing’ each of the WIDER recommendations and the supplementary recommendations would be an additional benefit to the development of this checklist. As it stands, it is unclear which of the four WIDER recommendations is the most important for the reporting of behavior change interventions. For example, proponents of theory-informed interventions might argue that category two (*i.e.*, intervention development, change techniques used, casual processes targeted) is the most important to report; without a detailed understanding of the change processes targeted and a comprehensive outline of the intervention development, replication of the intervention may be unlikely, the ‘active ingredients’ of the intervention may be difficult to identify, and the theory underlying the intervention subsequently cannot be tested. The WIDER recommendations and the supplementary recommendations should be weighted to reflect the degree of importance, which has not been rigorously reflected upon or studied.

## Conclusions

Ultimately, the guiding philosophy behind the WIDER Recommendations Checklist was the mantra: ‘Is this replicable?’ Through the application of the checklist in four systematic reviews, we found a high degree of variability in the reporting of KT interventions, which illuminates the difficulties faced in intervention replicability. We often felt a strong pull to give authors the benefit of the doubt when it came to assessing the quality of their reporting of BCIs; however, we believe that it is crucial that the information required by the WIDER recommendations is public and easily accessible in order to understand, replicate, and evaluate KT interventions. Missing information potentially points to methodological flaws and indicates the limitations of current scientific practice [5]. In the age of open access and online journals with unlimited ‘space’ for additional files to supplement research articles, what is the rationale for leaving information out? As of 2011, journals such as Addiction and Implementation Science require detailed descriptions of BCIs based on the WIDER recommendations framework in order to be published; therefore, it is important to be able to measure and compare the ability of studies to meet these recommendations. We believe that this checklist is one way to facilitate the measurement and comparison between a variety of KT intervention studies.

## Abbreviations

BCI: Behavior change interventions; KT: Knowledge translation; CONSORT: Consolidated standards of reporting trials; RCT: Randomized controlled trial; WIDER: Workgroup for intervention development and evaluation research.

## Competing interests

The authors declare that there are no competing interests.

## Authors’ contributions

LA conceptualized the checklist and coordinated the studies that facilitated the development of the checklist. SDS conceptualized the systematic reviews and secured study funding from the Canadian Institutes for Health Research (CIHR) and the Faculty of Nursing at the University of Alberta. She designed and led the systematic reviews. MA participated in the second stage of checklist development. DA participated in the third stage of checklist development. All authors contributed to manuscript drafts and reviewed the final manuscript.

## Authors’ information

We would like to acknowledge the organizations that provide research personnel funding for members of our research team. SDS holds a CIHR New Investigator Award and Population Health Investigator Award from the Alberta Heritage Foundation for Medical Research (now Alberta Innovates – Health Solutions). MA is a PhD student in the Faculty of Nursing and currently holds a Canadian Child Health Clinician-Scientist (CCHCSP) Doctoral Award, an Alberta Registered Nurses Educational Trust (ARNET) President’s Scholarship (Doctoral level), and a Doctoral Recruitment Scholarship (Faculty of Nursing, Univesity of Alberta). She would also like to thank the Women & Children’s Health Research Institute (WCHRI) for their support for her PhD research. DA is an undergraduate student in the Faculty of Nursing Bilingual Program and currently holds a CIHR Health Professional Student Research Award Designation, an Alberta Innovates – Health Solutions Summer Studentship, a Women & Children’s Health Research Institute (WCHRI) Summer Studentship, and a Faculty of Nursing Summer Studentship.

## Supplementary Material

Additional file 1 Improving reporting of behavioural interventions: WIDER Consensus Statement. Click here for file

Additional file 2: Table S2 WIDER Recommendations Checklist, Phase One. Click here for file

Additional file 3: Table S3 WIDER Recommendations Checklist, Phase Two. Click here for file

Additional file 4: Table S4 WIDER Checklist Development, Phase Three. Click here for file
