# Supplementary material for: Development of a checklist to assess the quality of reporting of knowledge translation interventions using the Workgroup for Intervention Development and Evaluation Research (WIDER) recommendations
Source: Implement Sci. 2013 May 16;8:52. doi: 10.1186/1748-5908-8-52 (PMC3661354; doi:10.1186/1748-5908-8-52)
Supplement: Additional file 1 — Improving reporting of behavioural interventions: WIDER Consensus Statement. [file 1748-5908-8-52-S1.pdf]

Improving reporting of behavioural interventions: WIDER Consensus Statement

Health-related behaviour patterns are critical to individual and public health. [1] Such behaviours, including smoking, alcohol consumption, eating, physical activity and adherence to medication also have major economic implications and often determine the effectiveness of medical treatments. Consequently considerable scientific effort has been developed to designing and evaluating the effectiveness of behavioural interventions designed to change health-related behaviour (BIs). Application of this work, including the adoption of effective BIs depends on accurate replication which, in turn, depends on detailed reporting of intervention characteristics e.g. techniques used, mode of delivery, intensity etc. [2] Ineffective BIs also need to be carefully reported so that design mistakes are not repeated and improvements can be tested in a systematic manner.

Guidelines on what should be included in reports of treatment and intervention trials (e.g., CONSORT) have shaped editorial policy and thereby enhanced and standardised details available in recent publications. [3,4] CONSORT guidance calls for “precise details of the interventions intended for each group and how and when they were actually administered”. (3, p.1192). Yet little progress has been made on reporting of intervention content.

A study of 80 consecutive articles featured in *Evidence-Based Medicine*, a journal which summarises research highly relevant to clinical practice found that elements of the intervention were missing in 41 (51%) cases. Reassuringly, however, the response rate to requests for further information was high for this

group of authors (88%) .[5] By contrast, a Cochrane review of audit and feedback interventions) was able to obtain required additional material about intervention content from only 27% authors contacted because of omissions in published reports [6]. Improvements are required both in the standardisation of reporting of BI content in published articles and in accessibility to additional information that can be made available to readers (including reviewers).

If readers are unable to obtain enough information to replicate a BI accurately then the reported design and evaluation work is lost to science. Readers are left knowing that something worked but not exactly *what* or *how*. Most BI evaluation reports do not provide sufficient detail to be enable replication. Detailed protocols need to be available only on request, but are often not forthcoming. To help remedy this situation, **W**orkgroup for **I**ntervention **D**evelopment and **E**valuation **R**esearch (WIDER) has developed brief guidance to journal editors. WIDER's aim is to improve the scientific reporting of BIs. We believe that CONSORT guidance need to be extended to allow better communication of the content of BIs and active controls. A complete version of the WIDER recommendations can be accessed through the EQUATOR website and at http://interventiondesign.co.uk/?page_id=9. A summary version is provided in table 1 and a list of signatories is provided below.

The recommendations address four issues. First, we recommend at that all editors should ensure that BI evaluations comply fully with the extended CONSORT statements for reporting of trials of non pharmacological treatments [7, 2] Providing standardised descriptions of BI characteristics in scientific articles would facilitate replication and comparison of BIs.

Second, readers (and reviewers) need to be aware of, and have access to, the principles that underpin researchers' development of BIs because this is critical to replication and systematic modification. In particular, evaluation papers should clarify; (1) what change processes were thought to be necessary to prompt a change in the specified behaviour, (2) how the intervention design was informed by theoretical considerations or models of causal or regulatory processes, and (3) what change techniques were included. The latter is especially important because such techniques constitute the unique (and potentially active) ingredients of a BI. Recent work has allowed the development of a taxonomy of discrete behaviour change techniques and also demonstrated that inclusion of specific change techniques is critical to BI effectiveness in particular domains. [8,9]

Third, even when BI evaluation reporting meets the standards outlined above, detailed information about materials and implementation cannot normally be included in the limited space available in scientific journals. This information must be included in protocols or manuals describing intervention implementation. Yet, as noted above, such manuals are often not available after a BI evaluation has been published. Consequently, we recommend that a detailed BI manuals are published (e.g., on a journal website) at the same time as any BI evaluation. This is already established practice for some journals. [10]

Finally, researchers need to know about the detailed content of active control groups (such as routine care comparisons). CONSORT guidance states that, "if the control treatment is usual care, authors should report all the components received by the control group" (7, p.288). In practice, this is rarely

achieved. Yet, observed intervention efficacy in trials that include active controls depends, in part, on the impact of change techniques employed in the active control condition. For example, an intervention that is effective when compared to poor routine care may fail to improve on state-of-the-art routine care. This is clearly demonstrated in a recent study which found that the content of routine care was a key factor in explaining the observed effectiveness of BI interventions designed to improve adherence to anti-retroviral drug regimens (de Bruin, Viechtbauer, Hospers, Schaalma, Kok, in press). If reviewers and researchers cannot accurately characterise (and compare) the content of active control conditions and so understand what effects these conditions are likely to have, they may misinterpret efficacy/ effectiveness data.

Adoption of the four WIDER recommendations alongside current CONSORT guidance will enhance the accessibility that researchers have to completed BI research and so greatly increase the impact that such research has on individual and public health.

1. Mokdad AH, Marks JS, Stroup DF, Gerberding JL. Actual causes of death in the United States. *JAMA* 2000; 291:1238-1245.

2. Davidson KW, Goldstein M, Kaplan RM, Kaufmann PG, Knatterund GL, et al. Evidence-based behavioral medicine: What is it and how do we achieve it? *Ann Beh Med* 2003; 26:161-171.

3. Moher D, Schulz KF, Altman DG. The CONSORT statement: Revised recommendations for improving the quality of reports of parallel group randomized trials. *Ann Int Med* 2001; 34:657-662.

4. Des Jarlais DC, Lyles C, Crepaz N. Improving the reporting quality of nonrandomized evaluations of behavioral and public health interventions: The TREND statement. *Am JPublic Health* 2004; 94:361-366.

5. Glasziou P, Meats E, Heneghan C, Shepperd S. What is missing from descriptions of treatment in trials and reviews? *BMJ* 2008; 336: 1472-1474.

6. Michie S, Johnston M, Francis J, Hardeman W, Eccles M. From theory to intervention: mapping theoretically derived behavioural determinants to behaviour change techniques. *Appl Psychol Int Rev* 2008; 57:660-680.

7. Boutron I, Moher D, Altman DG, Schulz KF, Ravaud P. for the CONSORT Group. Extending the CONSORT Statement to Randomized Trials of Nonpharmacologic Treatment: Explanation and Elaboration. *Ann Int Med,* 2008; 148:295-309.

8. Abraham C, Michie S. A taxonomy of behavior change techniques used in interventions. *Health Psychol* 2008; 27:379-387.

9. Michie S, Abraham C, Whittington C, McAteer J. Gupta S. Identifying Effective Techniques in Interventions: A meta-analysis and meta-regression. *Health Psychol* (in press).

10. West R. Providing full manuals and intervention descriptions: Addiction policy. *Addiction* 2008; 103:1411.

11. de Bruin M, Viechtbauer W, Hospers H, Schaalma H. Kok G. Standard Care Matters: Variability in standard care quality of HAART-adherence studies: Implications for the interpretation and comparison of intervention effects. *Health Psychol* (in press).

Table 1 Summary of WIDER Recommendations

1. Detailed Description of Interventions in Published Papers
Instructions to authors should specify that BI evaluations describe (1) characteristics of those delivering the intervention, (2) characteristics of the recipients, (3) the setting (e.g., worksite, time and place of intervention), (4) the mode of delivery (e.g., face-to-face) (5) the intensity (e.g., contact time), (6) the duration (e.g., number of sessions and their spacing over a given period), (7) adherence/ fidelity to delivery protocols and (8) a detailed description of the intervention content provided for each study group.

2. Clarification of Assumed Change Process and Design Principles
Instructions to authors should specify that BI evaluations describe (1) the intervention development, (2) the change techniques used in the intervention, and (3) the causal processes targeted by these change techniques in as much detail as possible, unless these details are easily available elsewhere (e.g., in a prior publication).

3. Access to Intervention Manuals/ Protocols
Before publishing a BI evaluation report, editors ask authors to submit a protocol or manual describing the BI or, alternatively, specify where such a manual can be easily and reliably accessed by readers.

4. Detailed Description of Active Control Conditions
Instructions to authors should specify that BI evaluations describe the content of active control groups in as much detail as is possible (e.g., the techniques used) in a similar manner to the description of the content of the intervention itself.

Footnote

The WIDER group includes

Professor Charles Abraham, University of Sussex, UK
and *Chair of WIDER*
Professor Dolores Albarracín, University of Illinois, USA.
Dr. Vera Araújo-Soares, Robert Gordon University, UK.
Dr. L. Kay Bartholomew, University of Texas, USA.
Professor Paul Bennett, University of Cardiff, UK
and editor of the *British Journal of Health Psychology*.
Professor Michael C. Costanza, Rushden, Northamptonshire UK
and *Statistical editor of Preventive Medicine*.
Professor David Crawford. Deakin University, Australia
and *Editor-in-Chief of the International Journal of Behavioral Nutrition and Physical Activity*
Professor Karina Davidson, Columbia University College of Physicians and Surgeons, USA and *Associate Editor of Health Psychology.*
Mr. Marijn de Bruin, University of Wageningen, The Netherlands.
Professor Nanne de Vries, University of Maastricht, The Netherlands.
Professor Martin Eccles Institute of Health and Society, Newcastle University
and *Co-Editor in Chief of Implementation Science.*
Professor Robbie Foy, Leeds Institute of Health Sciences, University of Leeds
and *Associate Editor of Implementation Science.*
Professor Gaston Godin, Université Laval, Canada.
Professor Robert Kaplan, Wasserman Distinguished Professor, UCLA, USA
and *Editor of Health Psychology.*
Professor Gerjo Kok, University of Maastricht.
Professor Blair Johnson, University of Connecticut, USA.
Professor Marie Johnston, University of Aberdeen, UK.
Professor Susan Michie, University College London.
Professor Rona Moss-Morris, University of Southampton, UK
and *Editor of Psychology & Health.*
Dr. Melissa Norton, BioMed Central LTD, Middlesex House, London
and *Editorial Director (Medicine),* BioMed Central
Dr. Jigisha Patel, BioMed Central LTD, Middlesex House, London
and Medical Editor of *BMC Public Health*
Dr. Gjalt-Jorn Peters, University of Maastricht, The Netherlands.
Dr. Rob Ruiter, University of Maastricht, The Netherlands
and *Associate Editor of Psychology & Health.*
Professor Herman Schaalma, University of Maastricht, The Netherlands
Professor Paschal Sheeran, University of Sheffield, UK.
Professor Lorraine Sherr, University College London.
Editor of AIDS Care, Psychology Health and Medicine
and *Editor of Vulnerable Children and Youth Studies*.
Dr. Falko Sniehotta, University of Aberdeen, UK.
Dr. Shaun Treweek, University of Dundee, UK.
Professor Joop van der Pligt, University of Amsterdam,The Netherlands

and *Editor of Health Psychology Review.*
Dr. Kavita Vedhara, University of Bristol, UK
and *Editor of the British Journal of Health Psychology.*
Professor Robert West, University College London, UK
and *Editor of Addiction.*
Professor Lucy Yardley, University of Southampton, UK
and *Editor of Psychology & Health*.
