# Supplementary material for: Development of a checklist to assess the quality of reporting of knowledge translation interventions using the Workgroup for Intervention Development and Evaluation Research (WIDER) recommendations
Source: Implement Sci. 2013 May 16;8:52. doi: 10.1186/1748-5908-8-52 (PMC3661354; doi:10.1186/1748-5908-8-52)
Supplement: Additional file 2: Table S2 — WIDER Recommendations Checklist, Phase One. [file 1748-5908-8-52-S2.doc]

Table 2: WIDER Recommendations Checklist, Phase One

| **First Author**  **(Year)** | **WIDER Recommendations to Improve Reporting**  **of the Content of Behaviour Change Interventions** | | | |
| --- | --- | --- | --- | --- |
|  | **Detailed Description of Intervention**  **(Y/N)** | **Clarification of Assumed Change Process and Design Principles**  **(Y/N)** | **Access to Intervention Manuals/ Protocols**  **(Y/N)** | **Detailed Description of Active Control Conditions**  **(Y/N)** |
| **Pharmacy Studies** | | | | |
| Hoffmann, W, et al. [1]  (2008) | N | N | Y | No active control |
| Hirsch, JD, et al. [2]  (2009) | N | N | N | No active control |
| Munroe, WP, et al. [3]  (1997) | N | N | N | No active control |
| Bracchi, RCG, et al. [4]  (2005) | N | N | N | No active control |
| Dualde, E, et al. [5]  (2009) | N | N | N | No control group |
| Airaksinen, M, et al. [6]  (1998) | N | N | N | No control group |
| Benrimoj, SI, et al. [7]  (2007) | N | Y | Y | No control group |
| Egen, V, et al. [8]  (2003) | N | N | N | No control group |
| Fjortoft, N, et al. [9]  (2003) | N | N | N | No control group |
| Fjortoft, N, et al. [10]  (2007) | N | N | N | No control group |
| Martin, BA, et al. [11]  (2010) | N | N | N | No control group |
| Brooks, VG, et al. [12]  (2001) | N | N | N | No active control |
| **Physiotherapy Studies** | | | | |
| Bekkering, GE, Hendricks, HJM, et al. [13]  (2005) | N | N | N | No active control |
| Bekkering, GE, van Tulder, MW, et al. [14]  (2005) | N | N | N | No active control |
| Hoeijenbos, M, et al. [15]  (2005) | N | N | N | No active control |
| Rebbeck, T, et al. [16]  (2006) | N | N | N | N |
| Stevenson, K, et al. [17]  (2006) | N | N | N | N |
| Kerssens, JJ, et al. [18]  (1999) | N | Y | Y | No active control |
| Brown, CJ et al. [19]  (2005) | N | N | N | No control group |
| Gross, DP, et al. [20]  (2009) | N | N | Y | No control group |
| Schreiber, J, et al. [21]  (2009) | N | N | N | No control group |
| **Physiotherapy & Occupational Therapy Studies** | | | | |
| Nikopoulou-Smyrni, P, et al. [22]  (2007) | N | N | N | N |
| Tripicchio, B, et al. [23]  (2009) | N | N | N | No control group |
| **Occupational Therapy Studies** | | | | |
| McCluskey, A, et al. [24]  (2005) | N | N | N | No control group |
| Hammond, A, et al. [25]  (2005) | N | N | N | No control group |
| McKenna, K, et al. [26]  (2005) | N | N | Y | No control group |
| Vachon, B, et al. [27]  (2009) | N | Y | N | No control group |
| **Dietetics Studies** | | | | |
| Banz, M, et al. [28]  (2004) | N | N | N | No active control |
| Brug, J, et al. [29]  (2007) | N | N | N | No active control |
| Johnson, ST, et al. [30]  (2007) | N | N | N | No active control |
| **Speech-Language Pathology Studies** | | | | |
| Pennington, L, et al. [31]  (2005) | N | N | N | No control group |
| Molfenter, SM, et al. [32]  (2009) | N | Y | N | No control group |

**References**

1. Hoffmann W, Herzog B, Muhlig S, Kayser H, Fabian R, Thomsen M, Cramer M, Fiβ T, Gresselmeyer D, Janhsen K: **Pharmaceutical care for migraine and headache patients: a community-based, randomized intervention.** *Ann Pharmacother* 2008, 42:1804-1813.
2. Hirsch JD, Rosenquist A, Best BM, Miller TA, Gilmer TP: **Evaluation of the first year of a pilot program in community pharmacy: HIV/AIDS medication therapy management for Medi-Cal beneficiaries.** *J Manag Care Pharm* 2009, 15:32-41.
3. Munroe WP, Kunz K, Dalmady-Israel C, Potter L, Schonfeld WH: **Economic evaluation of pharmacists involvement in disease management in a community pharmacy setting.** *Clin Ther* 1997, 19:113-123.
4. Bracchi RCG, Houghton J, Woods FJ, Thomas S, Smail SA, Routledge PA: **A distance-learning programme in pharmacovigilance linked to educational credits is associated with improved reporting of suspected adverse drug reactions via the UK yellow card scheme.** *Br J Clin Pharmacol* 2005, 60:221-223.
5. Dualde E, Faus MJ, Santonja FJ, Fernandez-Llimos F**: Effectiveness of a videoconference training course on implementing pharmacy services.** *Pharm World Sci* 2009, 31:638-642.
6. Airaksinen M, Ahonen R, Enlund H: **The “questions to ask about your medicines” campaign: an evaluation of pharmacists and the public’s response.** *Med Care* 1998, 36:422-427.
7. Benrimoj SL, Gilbert A, Quintrell N, de Almeida Neto AC: **Non-prescription medicines: a process for standards development and testing in community pharmacy.** *Pharm World Sci* 2007, 29:386-394.
8. Egen V, Hasford J: **Prevention of neural tube defects: effects of an intervention aimed at implementing the offician recommendations.** *Soz.- Präventivmed* 2003, 48:24–32.
9. Fjortoft N, Schwartz AH: **Evaluation of a pharmacy continuing education program: long-term learning outcomes and changes in practice behaviors.** *Am J Pharm Educ* 2003, 67:1-11.
10. Fjortoft N: **The effectiveness of commitment to change statements on improving practice behaviours following continuing pharmacy education.** *Am J Pharm Educ* 2007, 71:1-7.
11. Martin BA, Bruskiewitz RH, Chewning BA: **Effect of a tobacco cessation continuing professional education program on pharmacists’ confidence, skills, and practice-change behaviors.** *J Am Pharm Assoc* 2010, 50:9-16.
12. Brooks VG, Penick Brock T, Ahn J: **Do training programs work? an assessment of pharmacists activities in the field of chemical dependency.** *J Drug Educ* 2001, 31:153-169.
13. Bekkering GE, Hendriks HJM, van Tulder MW, Knol DL, Hoeijenbos M, Oostendorp RAB, Bouter LM: **Effect on the process of care of an active strategy to implement clinical guidelines on physiotherapy for low back pain: a cluster randomized controlled trial.** *Qual Saf Health Care* 2005, 14:107-112.
14. Bekkering GE, van Tulder MW, Hendriks EJM, Koopmanschap MA, Knol DL, Bouter LM, Oostendorp RAB: **Implementation of clinical guidelines on physical therapy for patients with low back pain: randomized trial comparing patient outcomes after a standard and active implementation strategy.** *Phys Ther* 2005, 85:544-555.
15. Hoeijenbos M, Bekkering T, Lamers L, Hendricks E, van Tulder M, Koopmanschap M: **Cost-effectiveness of an active implementation strategy for the Dutch physiotherapy guideline for low back pain**. *Health Policy* 2005, 75:85-98.
16. Rebbeck T, Maher CG, Refshauge KM: **Evaluating two implementation strategies for whiplash guidelines in physiotherapy: a cluster-randomised trial.** *Aust J Physiother* 2006, 52: 167-174.
17. Stevenson K, Lewis M, Hay E: **Does physiotherapy management of low back pain change as a result of an evidence-based educational programme.** *J Eval Clin Pract* 2004, 12:365-375.
18. Kerssens JJ, Sluijs EM, Verhaak PFM, Knibbe HJ, Hermans IMJ: **Educating patient educators: enhancing instructional effectiveness in physical therapy for low back patients**. *Patient Educ Couns* 1999, 37:165-176.
19. Brown CJ, Gottschalk M, Van Ness PH, Fortinsky RH, Tinetti ME: **Changes in physical therapy providers’ use of fall prevention strategies following a multicomponent behavioral change intervention.** *Phys Ther* 2005, 85(5):394-403.
20. Gross DP, Lowe A: **Evaluation of a knowledge translation initiative for physical therapists treating patients with work disability.** *Disabil Rehabil* 2009, 31:871-879.
21. Schreiber J, Stern P, Marchetti G, Provident I: **Strategies to promote evidence-based practice in pediatric physical therapy: a formative evaluation pilot project.** *Phys Ther* 2009, 89:918-933.
22. Nikopoulou-Smyrni P, Nikopoulos CK: **A new integrated model of clinical reasoning: development, description and preliminary assessment in patients with stroke.** *Disabil Rehabil* 2007, 29:1129-1138.
23. Tripicchio B, Bykerk K, Wegner C, Wegner J: **Increasing patient participation: the effects of training physical and occupational therapists to involve geriatric patients in the concerns-clarification and goal-setting processes.** *J Phys Ther Educ* 2009, 23:55-63.
24. McCluskey A, Lovarini M: **Providing education on evidence-based practice improved knowledge but did not change behavior: a before and after study.** *BMC Medical Education* 2005, 5:1-12.
25. Hammond A, Klompenhouwer P: **Getting evidence into practice: implementing a behavioural joint protection education programme for people with rheumatoid arthritis.** *Br J Occup Ther* 2005, 68:25-33.
26. McKenna K, Bennett S, Dierselhuis Z, Hoffmann T, Tooth L, McCluskey A: **Australian occupational therapists’ use of an online evidence-based practice database (OTseeker).** *Health Information and Libraries Journal* 2005, 22:205-214.
27. Vachon B, Durand MJ, LeBlanc J: **Using reflective learning to improve the impact of continuing education in the context of work rehabilitation**. *Adv in Health Sci Educ* 2010, 15:329-348.
28. Banz MF, Vineyard Most P, Banz WJ: **A workshop designed to educate dietetics professionals about the cardiovascular benefits of soyfoods.** *J Nutr Educ Behav* 2004, 36:103-104.
29. Brug J, Spikmans F, Aartsen C, Breedveld B, Bes R, Fereira I: **Training dietitians in basic motivational interviewing skills results in changes in their counseling style and lower saturated fat intakes in their patients*.*** *J Nutr Educ Behav* 2007, 39:8-12.
30. Johnson ST, Bates H, Fitzpatrick J, Marshall JD, Bell RC, McCargar L: **Promotion of physical activity by Canadian registered dietitians in daily practice.** *Hum Nutr Diet* 2007, 20:37-40.
31. Pennington L, Roddam H, Burton C, Russell I, Godfrey C, Russell D: **Promoting research use in speech and language therapy: a cluster randomized controlled trial to compare the clinical effectiveness and costs of two training strategies**. *Clin Rehabil* 2005, 19:387-397.
32. Molfentner SM, Ammoury A, Yeates EM, Steele CM: **Decreasing the knowledge-to-action gap through research-clinical partnerships in speech-language pathology.** *Can J Speech Lang Pathol Audiol* 2009, 33:82-88.
