# Supplementary material for: Development of a checklist to assess the quality of reporting of knowledge translation interventions using the Workgroup for Intervention Development and Evaluation Research (WIDER) recommendations
Source: Implement Sci. 2013 May 16;8:52. doi: 10.1186/1748-5908-8-52 (PMC3661354; doi:10.1186/1748-5908-8-52)
Supplement: Additional file 3: Table S3 — WIDER Recommendations Checklist, Phase Two. [file 1748-5908-8-52-S3.docx]

Table 3: WIDER Recommendations Checklist, Phase Two

| **First Author**  (KT Intervention) | **WIDER Recommendations to Improve Reporting of the Content of Behaviour Change Interventions** | | | | | | | | | | | | | | | | | | | |
| --- | --- | --- | --- | --- | --- | --- | --- | --- | --- | --- | --- | --- | --- | --- | --- | --- | --- | --- | --- | --- |
|  | **Recommendation 1:**  Detailed Description of Intervention | | | | | | | | **Recommendation 2:**  Clarification of Assumed Change Process and Design Principles | | | **Recommendation 3:**  Access to Intervention Manuals/ Protocols | **Recommendation 4:**  Detailed Description of Active Control Conditions | | | | | | | |
| **Randomized Controlled Trials** | | | | | | | | | | | | | | | | | | | | |
| **Adler et al.** [1]  (Professional: Educational meeting) |  |  |  |  |  |  |  |  |  |  |  |  |  |  |  |  |  |  |  |  |
| **^*^Cabana et al.** [2]  (Professional: Educational meeting “faculty training”) |  |  |  |  |  |  |  |  |  |  |  |  |  |  |  |  |  |  |  |  |
| **^*^Cabana et al.** [2]  (Professional: Educational meeting “physician training”) |  |  |  |  |  |  |  |  |  |  |  |  |  |  |  |  |  |  |  |  |
| **^*^Clark et al.** [3]  (Professional: Educational meeting “faculty training”) |  |  |  |  |  |  |  |  |  |  |  |  |  |  |  |  |  |  |  |  |
| **^*^Clark et al.** [3]  (Professional: Educational meeting “physician training”) |  |  |  |  |  |  |  |  |  |  |  |  |  |  |  |  |  |  |  |  |
| **^✓^Christakis et al.** [4]  (Professional: Reminders) |  |  |  |  |  |  |  |  |  |  |  |  |  |  |  |  |  |  |  |  |
| **^✓^Davis et al.** [5]  (Professional: Reminders) |  |  |  |  |  |  |  |  |  |  |  |  |  |  |  |  |  |  |  |  |
| **Epstein et al.** [6]  (Organizational – Provider Oriented: Clinical multidisciplinary team) |  |  |  |  |  |  |  |  |  |  |  |  |  |  |  |  |  |  |  |  |
| **Hillman et al.** [7]  (Professional: Audit & feedback) |  |  |  |  |  |  |  |  |  |  |  |  |  |  |  |  |  |  |  |  |
| **Hillman et al.** [7]  (Financial intervention - Provider Oriented: Provider incentives) |  |  |  |  |  |  |  |  |  |  |  |  |  |  |  |  |  |  |  |  |
| **^∞^Horbar et al.** [8]  (Professional: Audit & feedback) |  |  |  |  |  |  |  |  |  |  |  |  |  |  |  |  |  |  |  |  |
| **^∞^Horbar et al.** [8]  (Professional: Reminders) |  |  |  |  |  |  |  |  |  |  |  |  |  |  |  |  |  |  |  |  |
| **^∞^Horbar et al.** [8]  (Professional: Educational meeting) |  |  |  |  |  |  |  |  |  |  |  |  |  |  |  |  |  |  |  |  |
| **Johnston et al.** [9] (Professional: Educational meeting) |  |  |  |  |  |  |  |  |  |  |  |  |  |  |  |  |  |  |  |  |
| **Johnston et al.** [9] (Professional: Audit & feedback) |  |  |  |  |  |  |  |  |  |  |  |  |  |  |  |  |  |  |  |  |
| **^∆^Liaw et al.** [10]  (Professional: Educational meeting) |  |  |  |  |  |  |  |  |  |  |  |  |  |  |  |  |  |  |  |  |
| **^∆^Liaw et al.** [10]  (Professional: Educational material) |  |  |  |  |  |  |  |  |  |  |  |  |  |  |  |  |  |  |  |  |
| **^∆^Sulaiman et al.** [11] (Professional: Educational meeting) |  |  |  |  |  |  |  |  |  |  |  |  |  |  |  |  |  |  |  |  |
| **^∆^Sulaiman et al.** [11] (Professional: Educational material) |  |  |  |  |  |  |  |  |  |  |  |  |  |  |  |  |  |  |  |  |
| **Sanci et al.** [12]  (Professional: Educational meeting) |  |  |  |  |  |  |  |  |  |  |  |  |  |  |  |  |  |  |  |  |
| **Sanci et al.** [12]  (Professional: Educational material) |  |  |  |  |  |  |  |  |  |  |  |  |  |  |  |  |  |  |  |  |
| **^□^Tebb et al.** [13]  (Organizational – Provider Oriented: Clinical multidisciplinary team) |  |  |  |  |  |  |  |  |  |  |  |  |  |  |  |  |  |  |  |  |
| **Clinical Controlled Trials** | | | | | | | | | | | | | | | | | | | | |
| **Clarkson et al.** [14]  (Financial intervention – Provider: Fee for service) |  |  |  |  |  |  |  |  |  |  |  |  |  |  |  |  |  |  |  |  |
| **Clarkson et al.** [14] (Professional: Educational meeting) |  |  |  |  |  |  |  |  |  |  |  |  |  |  |  |  |  |  |  |  |
| **^□^Shafer et al.** [15]  (Organizational – Provider Oriented: Clinical multidisciplinary team) |  |  |  |  |  |  |  |  |  |  |  |  |  |  |  |  |  |  |  |  |
| **Controlled Before/After Studies** | | | | | | | | | | | | | | | | | | | | |
| **D'Alessandro et al.** [16] (Professional: Educational meeting) |  |  |  |  |  |  |  |  |  |  |  |  |  |  |  |  |  |  |  |  |
| **D’Allessandro et al.** [16]  (Professional: Educational material) |  |  |  |  |  |  |  |  |  |  |  |  |  |  |  |  |  |  |  |  |
| **Edwards et al.** [17]  (Professional: Educational outreach visit) |  |  |  |  |  |  |  |  |  |  |  |  |  |  |  |  |  |  |  |  |
| **^∞^Rogowski et al.** [18]  (Organizational – Provider Oriented: Clinical multidisciplinary team) |  |  |  |  |  |  |  |  |  |  |  |  |  |  |  |  |  |  |  |  |
| **Naimoli et al.** [19]  (Professional: Educational meeting “Train the trainer”) |  |  |  |  |  |  |  |  |  |  |  |  |  |  |  |  |  |  |  |  |
| **Naimoli et al.** [19]  (Professional: Educational meeting “In-service training”) |  |  |  |  |  |  |  |  |  |  |  |  |  |  |  |  |  |  |  |  |
| **Naimoli et al.** [19]  (Professional: Educational outreach visit) |  |  |  |  |  |  |  |  |  |  |  |  |  |  |  |  |  |  |  |  |
| **Smabrekke et al.** [20]  (Professional: Educational meeting) |  |  |  |  |  |  |  |  |  |  |  |  |  |  |  |  |  |  |  |  |
| **Smabrekke et al.** [20] (Professional: Educational material) |  |  |  |  |  |  |  |  |  |  |  |  |  |  |  |  |  |  |  |  |
| **Tucker et al.** [21]  (Professional: Educational meeting) |  |  |  |  |  |  |  |  |  |  |  |  |  |  |  |  |  |  |  |  |

**Legend**

Dark Grey Shading = Recommended supplementary recommendation met

Light Grey Shading = Same study (with multiple interventions)

Multiple publications using the same data grouped together by with a symbol (i.e., *, ✓,∞,∆,□)

**References**

1. Adler MD, Vozenilek JA, Trainor JL, Eppich WJ, Wang EE, Beaumont JL, Aitchison PR, Erikson T, Edison M, McGaghie WC: **Development and evaluation of a simulation-based pediatric emergency medicine curriculum.** *Acad Med* 2009, 84(7):935-941.
2. Cabana MD, Slish KK, Evans D, Mellins RB, Brown RW, Lin X, Kaciroti N, Clark NM: **Impact of physician asthma care education on patient outcomes**. *Pediatrics* 2009, 117(6):2149-2157.
3. Clark NM, Cabana M, Kaciroti N, Gong M, Sleeman K: **Long-term outcomes of physician peer-teaching.** *Clin Pediatr* 2008, 47(9):883-890.
4. Christakis DA, Zimmerman FJ, Wright JA, Garrison MM, Rivara FR, Davis RL: **A randomized controlled trial of point-of-care evidence to improve the antibiotic prescribing practices for otitis media in children**. *Pediatrics* 2001, 107(2):e15.
5. Davis RL, Wright J, Chalmers F, Levenson L, Brown JC, Lozano P, Christakis DA: **A cluster randomized clinical trial to improve prescribing patters in ambulatory pediatrics**. *PloS Clinical Trials* 2007, 2(5):e25.
6. Epstein JN, Rabiner DR, Johnson DE, Fitzgerald DP, Chrisman A, Erkanli A, Sullivan KK, March JS, Margolis P, Norton EC, Conners CK: **Improving attention-deficit/hyperactivity disorder treatment outcomes through use of a collaborative consultation treatment service by community-based pediatricians**. *Arch Pediatr Adolesc Med* 2007, 161(9):835-840.
7. Hillman AI, Ripley K, Goldfarb N, Weiner J, Nuamah I, Lusk E: T**he use of physician financial incentives and feedback to improve pediatric preventive care in medicaid managed care**. *Pediatrics* 1999, 104(4):931-935.
8. Horbar JD, Carpenter JH, Buzas J, Soll, RF, Suresh G, Bracken MB, Leviton LC, Plsek PE, Sinclair JC: **Collaborative quality improvement to promote evidence based surfactant for preterm infants: A cluster randomized trial.** *BMJ* 2004, 329(7473):1004-1007.
9. Johnson CC, Gagnon A, Rennick J, Rosmus C, Patenaude H, Ellis J, Shapiro C. Filion F, Ritchie J, Byron J: **One-on-one coaching to improve pain assessment and management practices of pediatric nurses**. *JPN* 2007, 22(6):467-478.
10. Liaw S, Sulaiman NB, Barton CA, Chondros P, Harris CA, Sawyer S, Dharmage S: **An interative workshop plus locally adapted guidelines can improve General Practitioners asthma management and knowledge: A cluster randomised trial in the Australian setting**. *BMC Family Practice* 2008, 9(22).
11. Sulaiman ND, Barton CA, Liaw, S, Harris CA, Sawyer SM, Abramson MJ, Robertson C, Dharmage SC: **Do small group workshops and locally adapted guidelines improve asthma patients’ health outcomes? A cluster randomized controlled trial.** *Family Practice* 2010, 27:246-254.
12. Sanci LA, Coffey CMM, Veit FCM, Carr-Gregg M, Patton GC, Day N, Bowes G: **Evaluation of the effectiveness of an educational intervention for general practitioners in adolescent health care: Randomised controlled trial.** *BMJ* 2000, 320:224-229.
13. Tebb KP, Wibbelsman C, Neuhaus JM, Shafer M: **Screening asymptomatic chlamydia infections among sexually active adolescent girls during pediatric urgent care.** *Arch Pediatr & Adolesc Med* 2009, 163(6):559-564.
14. Clarkson JE, Turner S, Grimshaw JM, Ramsay CR, Johnston M, Scott A, Bonetti D, Tilley CJ, Maclennan G, Ibbetson R, MacPherson LMD, Pitts NB: **Changing clinicians’ behaviour: A randomized controlled trial of fees and education**. *J Dent Res* 2008, 87(7):640-644.
15. Shafer MB, Tebb KP, Pantell RH, Wibbelsman CJ, Neuhaus JM, Tipton AC, Kunin SB, KO TH, Schweppe DM, Bergman DA: **Effect of a clinical practice improvement intervention on chlamydial screening among adolescent girls**. *JAMA* 2002, 288(22):2846-2852.
16. D’Allessandro DM, Kreiter CD, Peterson MW: **An evaluation of information-seeking behaviours of general pediatricians**. *Pediatrics* 2004, 113(1):64-69.
17. Edwards H, Walsh A, Courtney M, Monaghan S, Wilson J, Young J: **Promoting evidence-based childhood fever management through a peer education programme based on the theory of planned behaviour**. *Journal Clin Nurs*2007, 16:1966-1979.
18. Rogowski JA, Horbar JA, Plsek PE, Baker LS, Deterding J, Edwards WH, Hocker J, Kantak AD, Lewallen P, Lewis W, Lewit E, McCarroll CJ, Mujsce D, Payne NR, Shiono P, Soll RF, Leahy K: **Economic implications of neonatal intensive care unit collaborative quality improvement**. *Pediatrics* 2001, 107(1):23-29.
19. Naimoli JF, Rowe AK, Lyaghfouri A, Larbi R, Lamrani LA: **Effect of the integrated management of childhood illness strategy on health care quality in Morocco**. *Int J Qual Health Care* 2006, 18(2):134-144.
20. Smabrekke L, Berild D, Giaever A, Myrbakk T, Fuskevag A, Ericson JU, Flaegstad T, Olsvik O, Ringertz SH: **Educational intervention for parents and healthcare providers leads to reduced antibiotic use in acute otitis media**. *Scand J Infect Dis* 2002, 34:657-659.
21. Tucker S, Derscheid D, Odegarden S, Olson ME: **Evidence-based training for enhancing psychiatric nurses’ child behaviour management skills**. *J Nurses Staff Dev* 2008, 24(2):75-85.
