# Supplementary material for: Development of a checklist to assess the quality of reporting of knowledge translation interventions using the Workgroup for Intervention Development and Evaluation Research (WIDER) recommendations
Source: Implement Sci. 2013 May 16;8:52. doi: 10.1186/1748-5908-8-52 (PMC3661354; doi:10.1186/1748-5908-8-52)
Supplement: Additional file 4: Table S4 — WIDER Checklist Development, Phase Three. [file 1748-5908-8-52-S4.docx]

Table 4: WIDER Checklist Development, Phase Three

| **First Author (Year)** | **WIDER Recommendations to Improve Reporting of the**  **Content of Behaviour Change Interventions** | | | | | | | | | | | | | | | | | | | | | | | | |
| --- | --- | --- | --- | --- | --- | --- | --- | --- | --- | --- | --- | --- | --- | --- | --- | --- | --- | --- | --- | --- | --- | --- | --- | --- | --- |
|  | **Recommendation #1**  Detailed Description of Intervention | | | | | | | |  | **Recommendation #2**  Clarification of Assumed Change Process and Design Principles | | |  | **Recommendation #3**  Access to Intervention Manuals/Protocols |  | **Recommendation #4**  Detailed Description of  Active Control Conditions | | | | | | | | | |
| **Physiotherapy Studies** | | | | | | | | | | | | | | | | | | | | | | | | | |
| *Bekkering, GE, Hendricks, HJM, et al.  (2005) [1] | 1 | 2 | 3 | 4 | 5 | 6 | 7 | 8 |  | A | B | C |  | NO |  | 1 | 2 | 3 | 4 | | 5 | | 6 | 7 | 8 |
| *Bekkering, GE, van Tulder MW, et al. (2005) [2] | 1 | 2 | 3 | 4 | 5 | 6 | 7 | 8 |  | A | B | C |  | NO |  | 1 | 2 | 3 | 4 | | 5 | | 6 | 7 | 8 |
| *Hoeijenbos, M, et al. (2005) [3] | 1 | 2 | 3 | 4 | 5 | 6 | 7 | 8 |  | A | B | C |  | NO |  | 1 | 2 | 3 | 4 | | 5 | | 6 | 7 | 8 |
| Rebbeck, T, et al. (2006) [4] | 1 | 2 | 3 | 4 | 5 | 6 | 7 | 8 |  | A | B | C |  | NO |  | 1 | 2 | 3 | 4 | | 5 | | 6 | 7 | 8 |
| Stevenson, K, et al. (2006) [5] | 1 | 2 | 3 | 4 | 5 | 6 | 7 | 8 |  | A | B | C |  | NO |  | 1 | 2 | 3 | 4 | | 5 | | 6 | 7 | 8 |
| Kerssens, JJ, et al. (1999) [6] | 1 | 2 | 3 | 4 | 5 | 6 | 7 | 8 |  | A | B | C |  | YES |  | No control group | | | | | | | | | |
| Brown, CJ et al.(2005) [7] | 1 | 2 | 3 | 4 | 5 | 6 | 7 | 8 |  | A | B | C |  | NO |  | No control group | | | | | | | | | |
| Gross, DP, et al. (2009) [8] | 1 | 2 | 3 | 4 | 5 | 6 | 7 | 8 |  | A | B | C |  | YES |  | No control group | | | | | | | | | |
| Schreiber, J, et al. (2009) [9] | 1 | 2 | 3 | 4 | 5 | 6 | 7 | 8 |  | A | B | C |  | NO |  | No control group | | | | | | | | | |
| **Physiotherapy & Occupational Therapy Studies** | | | | | | | | | | | | | | | | | | | | | | | | | |
| Nikopoulou-Smyrni, P, et al. (2007) [10] | 1 | 2 | 3 | 4 | 5 | 6 | 7 | 8 |  | A | B | C |  | NO |  | 1 | 2 | 3 | | 4 | | 5 | 6 | 7 | 8 |
| Tripicchio, B, et al. (2009) [11] | 1 | 2 | 3 | 4 | 5 | 6 | 7 | 8 |  | A | B | C |  | NO |  | No control group | | | | | | | | | |
| **Occupational Therapy Studies** | | | | | | | | | | | | | | | | | | | | | | | | | |
| McCluskey, A, et al. (2005) [12] | 1 | 2 | 3 | 4 | 5 | 6 | 7 | 8 |  | A | B | C |  | NO |  | No control group | | | | | | | | | |
| Hammond, A, et al. (2005) [13] | 1 | 2 | 3 | 4 | 5 | 6 | 7 | 8 |  | A | B | C |  | NO |  | No control group | | | | | | | | | |
| McKenna, K, et al. (2005) [14] | 1 | 2 | 3 | 4 | 5 | 6 | 7 | 8 |  | A | B | C |  | YES |  | No control group | | | | | | | | | |
| Vachon, B, et al. (2009) [15] | 1 | 2 | 3 | 4 | 5 | 6 | 7 | 8 |  | A | B | C |  | NO |  | No control group | | | | | | | | | |
| **Speech-Language Pathology Studies** | | | | | | | | | | | | | | | | | | | | | | | | | |
| Pennington, L, et al. (2005) [16] | 1 | 2 | 3 | 4 | 5 | 6 | 7 | 8 |  | A | B | C |  | NO |  | No control group | | | | | | | | | |
| Molfenter, S, et al. (2009) [17] | 1 | 2 | 3 | 4 | 5 | 6 | 7 | 8 |  | A | B | C |  | NO |  | No control group | | | | | | | | | |

**Legend**

* = articles related to same research study

Grey = supplementary recommendation met

White = supplementary recommendation not met

Recommendations #1 & #4:

1) Characteristics of those delivering the intervention/control condition

2) Characteristics of the recipients

3) Setting

4) Mode of delivery

5) Intensity

6) Duration

7) Adherence/ fidelity to delivery protocols

8) Detailed description of the intervention/control condition

Recommendation #2:

A) Intervention development described

B) Change techniques employed in intervention identified and described

C) Causal processes targetted by change techniques identified and described

**References**

1. Bekkering GE, Hendriks HJM, van Tulder MW, Knol DL, Hoeijenbos M, Oostendorp RAB, Bouter LM: **Effect on the process of care of an active strategy to implement clinical guidelines on physiotherapy for low back pain: a cluster randomized controlled trial.** *Qual Saf Health Care* 2005, 14:107-112.
2. Bekkering GE, van Tulder MW, Hendriks EJM, Koopmanschap MA, Knol DL, Bouter LM, Oostendorp RAB: **Implementation of clinical guidelines on physical therapy for patients with low back pain: randomized trial comparing patient outcomes after a standard and active implementation strategy.** *Phys Ther* 2005, 85:544-555.
3. Hoeijenbos M, Bekkering T, Lamers L, Hendricks E, van Tulder M, Koopmanschap M: **Cost-effectiveness of an active implementation strategy for the Dutch physiotherapy guideline for low back pain**. *Health Policy* 2005, 75:85-98.
4. Rebbeck T, Maher CG, Refshauge KM: **Evaluating two implementation strategies for whiplash guidelines in physiotherapy: a cluster-randomised trial.** *Aust J Physiother* 2006, 52: 167-174.
5. Stevenson K, Lewis M, Hay E: **Does physiotherapy management of low back pain change as a result of an evidence-based educational programme.** *J Eval Clin Pract* 2004, 12:365-375.
6. Kerssens JJ, Sluijs EM, Verhaak PFM, Knibbe HJ, Hermans IMJ: **Educating patient educators: enhancing instructional effectiveness in physical therapy for low back patients**. *Patient Educ Couns* 1999, 37:165-176.
7. Brown CJ, Gottschalk M, Van Ness PH, Fortinsky RH, Tinetti ME: **Changes in physical therapy providers’ use of fall prevention strategies following a multicomponent behavioral change intervention.** *Phys Ther* 2005, 85(5):394-403.
8. Gross DP, Lowe A: **Evaluation of a knowledge translation initiative for physical therapists treating patients with work disability.** *Disabil Rehabil* 2009, 31:871-879.
9. Schreiber J, Stern P, Marchetti G, Provident I: **Strategies to promote evidence-based practice in pediatric physical therapy: a formative evaluation pilot project.** *Phys Ther* 2009, 89:918-933.
10. Nikopoulou-Smyrni P, Nikopoulos CK: **A new integrated model of clinical reasoning: development, description and preliminary assessment in patients with stroke.** *Disabil Rehabil* 2007, 29:1129-1138.
11. Tripicchio B, Bykerk K, Wegner C, Wegner J: **Increasing patient participation: the effects of training physical and occupational therapists to involve geriatric patients in the concerns-clarification and goal-setting processes.** *J Phys Ther Educ* 2009, 23:55-63.
12. McCluskey A, Lovarini M: **Providing education on evidence-based practice improved knowledge but did not change behavior: a before and after study.** *BMC Medical Education* 2005, 5:1-12.
13. Hammond A, Klompenhouwer P: **Getting evidence into practice: implementing a behavioural joint protection education programme for people with rheumatoid arthritis.** *Br J Occup Ther* 2005, 68:25-33.
14. McKenna K, Bennett S, Dierselhuis Z, Hoffmann T, Tooth L, McCluskey A: **Australian occupational therapists’ use of an online evidence-based practice database (OTseeker).** *Health Information and Libraries Journal* 2005, 22:205-214.
15. Vachon B, Durand MJ, LeBlanc J: **Using reflective learning to improve the impact of continuing education in the context of work rehabilitation**. *Adv in Health Sci Educ* 2010, 15:329-348.
